# MicroRNA-18a-5p regulates hepatic lipid accumulation in response to high-fat diet

**DOI:** 10.3389/fphys.2025.1661428

**Published:** 2025-09-04

**Authors:** Giuseppe Petito, Nunzia Magnacca, Arianna Cuomo, Maria Ventriglia, Angelo Fusco, Massimo Venditti, Sara Falvo, Nicoletta Potenza, Antonia Lanni, Federica Cioffi, Rosalba Senese

**Affiliations:** ^1^ Department of Environmental, Biological and Pharmaceutical Sciences and Technologies, University of Campania “Luigi Vanvitelli”, Caserta, Italy; ^2^ Department of Experimental Medicine, University of Campania “Luigi Vanvitelli”, Naples, Italy; ^3^ Department of Science and Technologies, University of Sannio, Benevento, Italy

**Keywords:** miRNA, ER stress, autophagy, apoptosis, fats, liver, triglyceride

## Abstract

**Introduction:**

Metabolic Dysfunction-Associated Steatotic Liver Disease (MASLD), formerly known as Nonalcoholic Fatty Liver Disease (NAFLD), is characterized by hepatic lipid accumulation, inflammation, and progressive liver injury, potentially leading to steatohepatitis, cirrhosis, and hepatocellular carcinoma (HCC). Central to MASLD pathogenesis are dysregulated lipid metabolism and unresolved endoplasmic reticulum (ER) stress, with sterol regulatory element-binding protein 1 c (SREBP1c) and the protein kinase RNA-like ER kinase (PERK) -eukaryotic initiation factor 2 alpha (eIF2α) signaling pathway playing key roles. This study investigates the regulatory role of microRNA-18a-5p (miR-18a-5p) in lipid accumulation during MASLD induced by a high-fat diet (HFD).

**Methods:**

Experiments were performed on male Wistar rats fed either a standard or high-fat diet to induce MASLD. In addition, HepG2 cells were treated with fatty acids to establish an in vitro model of MASLD.

**Results:**

In HFD fed rats, miR-18a-5p was significantly downregulated, coinciding with increased SREBP1c expression, PERK pathway activation, hepatic lipid accumulation, apoptosis, and impaired autophagy flux. A similar pattern was observed in fatty acid-treated HepG2 cells, confirming the translational relevance of the findings. Notably, miR-18a-5p overexpression reduced lipid accumulation, attenuated ER stress, restored autophagy, and suppressed apoptosis, in both *in vivo* and *in vitro* models.

**Conclusion:**

These results identify miR-18a-5p as a key regulator of lipid homeostasis and ER stress in MASLD, suggesting its potential as a novel therapeutic target. Understanding such molecular mechanisms is crucial for developing effective strategies against this increasingly prevalent liver disease.

## 1 Introduction

In recent decades, the global rise in obesity has contributed to a surge in metabolic disorders. Among these, Metabolic Dysfunction-Associated Steatotic Liver Disease (MASLD), formerly known as Nonalcoholic Fatty Liver Disease (NAFLD), is characterized by hepatic steatosis and at least one cardiometabolic risk factor (Lazarus et al., 2024; Yanai et al., 2023; Younossi et al., 2023).

Excessive liver lipid accumulation in MASLD initiates an inflammatory response and progressive liver damage, which can lead to more severe conditions, including Metabolic Dysfunction-Associated Steatohepatitis (MASH) cirrhosis, and hepatocellular carcinoma (HCC) (Chalasani et al., 2018; Cioffi et al., 2022; Younossi et al., 2018). High-fat diets (HFDs) are a primary contributor, promoting lipid accumulation and related liver damage (Lian et al., 2020).

Despite the increasing prevalence of MASLD, effective treatments remain limited, highlighting the importance of elucidating its molecular mechanisms. Central to lipid regulation in the liver are sterol regulatory element-binding proteins (SREBPs), especially sterol regulatory element-binding protein 1 c (SREBP1c), which controls hepatic lipogenesis by activating genes involved in fatty acid and triglyceride synthesis (Ferré et al., 2021; Horton et al., 2002). Dysregulation of SREBP1c is implicated in MASLD, Type 2 Diabetes Mellitus (T2DM), and atherosclerosis (Li et al., 2023). In transgenic mice, overexpression of SREBP1c increases lipogenesis, and induces hepatic steatosis, while its deletion reduces triglyceride levels (Moon et al., 2012; Shimano et al., 1997). HFDs can elevate hepatic SREBP1c expression, linking hyperinsulinemia to increased lipid synthesis and progression of fatty liver disease (Ascencio et al., 2004; Buettner et al., 2006). Clinical evidence additionally reveals elevated *de novo* lipogenesis in MASLD patients, often associated with persistent SREBP1c activation (Lambert et al., 2014).

Research increasingly highlights the role of Endoplasmic Reticulum (ER) stress in the pathogenesis and progression of MASLD (Lebeaupin et al., 2018). The ER controls protein folding, lipid metabolism, and calcium balance, but lipid overload can disrupt its function, leading to ER stress (Mota et al., 2016). In response, the Unfolded Protein Response (UPR) attemps to restore homeostasis by enhancing protein folding, reducing translation, and promoting autophagy (Almanza et al., 2019; Hetz, 2012; Liu and Green, 2019). However, prolonged ER stress can lead to hepatocyte apoptosis, inflammation, and liver damage, exacerbating MASLD (Sano and Reed, 2013; Zou and Qi, 2020). ER stress and UPR activation upregulate SREBP1c, enhancing lipid biosynthesis and accumulation (Ferré et al., 2021; Kammoun et al., 2009; Sethi et al., 2017). Activation of the protein kinase RNA-like ER kinase (PERK) - eukaryotic initiation factor 2 alpha (eIF2α) pathway, a UPR branch, also promotes lipid accumulation by upregulating SREBP1c and sterol regulatory element-binding protein 2 (SREBP2) in hepatocytes (Lauressergues et al., 2010). Conversely, the mature nuclear forms of SREBPs, nSREBP1, bind to the promoter of PERK, influencing UPR activation ER and autophagy (Hu et al., 2020) (Supplementary Figure S1).

In addition to transcriptional regulation, post-transcriptional mechanisms such as microRNAs (miRNAs) are increasingly recognized for their role in lipid metabolism and MASLD pathophysiology (Gjorgjieva et al., 2019; López-Pastor et al., 2020). miRNAs are small non-coding RNA molecules that regulate gene expression by targeting mRNAs for degradation or translational repression. Several miRNAs, including microRNA-18a-5p (miR-18a-5p), have been linked to lipid metabolism and are dysregulated in metabolic disorders (Huang et al., 2020). Reduced levels of miR-18a-5p are associated with metabolic syndrome and T2DM, suggesting a role in insulin signaling and glucose homeostasis (Zhou et al., 2020). Furthermore, studies on highly lung-metastatic breast cancer cell lines, where reduced expression of miR-18a-5p correlates with concomitant overexpression of SREBP1, have demonstrated that SREBP1 is a direct target of miR-18a-5p (Zhang et al., 2019).

In light of these findings, and following an initial serum miRNA screening that revealed significant downregulation of miR-18a-5p in HFD-treated rats compared to controls on a standard diet, this study examines the variations of hepatic expression of miR-18a-5p and its potential regulatory relationship with SREBP1c and key components of the UPR signaling pathways. To validate the role of the miR-18a-5p in modulating lipid accumulation and ER stress, HepG2 cells were transfected with a miR-18a-5p mimic and treated with an oleate/palmitate mixture.

## 2 Materials and methods

### 2.1 Animals and animal care

All animals received humane care according to the criteria outlined in the Guide for the Care and Use of Laboratory Animals prepared by the National Academy of Sciences and published by the National Institutes of Health. All animal protocols were approved by the Organism in charge of animal welfare (OBPA) of the University of Campania “Luigi Vanvitelli” (Caserta, Italy) and the Italian Minister of Health (Permit Number: 704/2016-PR). Every effort was made to minimize animal pain and suffering. All methods were reported in accordance with the ARRIVE guidelines and have been carried out in accordance with EU Directive 2010/63/EU for animal experiment. The minimum sample size (n = 6) was determined using a power analysis performed with G*Power software (developed by the University of Düsseldorf: http://www.gpower.hhu.de/), as required by the legal authority responsible for approving *in vivo* experiments in Italy. Male Wistar Rats (8-weeks-old) purchased from Charles River (Charles River, Wilmington, MA, United States) were individually caged in a temperature-controlled room at 28 °C (thermoneutrality temperature for rats) under a 12-h light/12-h dark cycle. The animals were housed individually to allow precise monitoring of food intake, which was essential for our experimental design. Furthermore, to help mitigate potential stress associated with single housing, environmental enrichment was provided in the form of aspen balls and aspen sticks (Inotiv Inc., West Lafayette, Indiana, United States), which are safe, non-toxic wooden items that promote natural gnawing behavior and encourage interaction with the environment. Before the commencement of the study, a commercial mash (Charles River, Wilmington, MA, United States) was available *ad libitum* and the animals had free access to water. At the start of the study (day 0), after 7 days of acclimatization to thermoneutrality, the rats were divided into two groups of 6 (n = 6) and treated for 5 weeks as follows.

• First group (N), received a standard natural-ingredient diet for 5 weeks (total metabolizable percentage of energy: 60.4 carbohydrates, 29 proteins, 10.6 fat J J−1; 15.88 kJ gross energy g−1; Global Diet 2018S from Harlan Teklad);
• Second group (HFD), received a high-fat diet for 5 weeks (280 g of diet supplemented with 395 g of lyophilized lamb meat [Liomellin, Milan, Italy], 120 g cellulose [Sigma-Aldrich, St. Louis, MO, United States], 20 g mineral mix [ICN Biomedical, Solon, OH], 7 g vitamin mix [ICN Biomedical, Solon, OH], and 200 g low-salt butter [Lurpak, Holstrebo, Denmark]; total metabolizable percentage of energy: 21 carbohydrates, 29 proteins, 50 fat J J−1; 19.85 kJ gross energy g−1).

Body weight of each animal was recorded twice weekly in the morning. Animals were monitored for any clinical abnormalities during each weight measurement. Food intake was measured throughout the entire experimental period. The amount of food consumed (g) by each rat was calculated by subtracting the weight of uneaten food from the initial weight of the provided food. Upon completion of the treatment, the rats were anesthetized and euthanized by decapitation. Blood was collected and centrifuged at 2.000 × g to obtain the serum, which was then aliquoted and stored at −20 °C. The liver was excised, weighed, immediately frozen in liquid nitrogen, and stored at −80 °C for subsequent processing.

### 2.2 Metabolic parameters

The serum concentrations of total cholesterol, triglycerides, and GPT-ALT were measured using colorimetric enzymatic methods with commercially available kits (SGM Italia, Rome, Italy), as previously published (Petito et al., 2021). For the Intraperitoneal Glucose Tolerance Test (IPGTT), rats were fasted for 6 h. A baseline (0-min) blood sample was collected via a small tail clip, followed by an intraperitoneal injection of glucose (2 g/kg body weight in strile solution). Blood samples were then taken at 15, 20, 30, 90 and 180 min after injection. For the Insulin Tolerance Test (ITT), rats were fasted for 6 h and subsequently injected intraperitoneally with insulin (Humalog, 2 units/kg body weight in sterile saline) (Eli Lilly, Utrecht, Nederland). Blood samples were collected at 10, 15, 20, 30, and 45 min post injection.

### 2.3 Immunoassay for 8-hydroxy-2′-Deoxyguanosine (8-OHdG)

A competitive ELISA for 8-OHdG was performed using a DNA/RNA Oxidative Damage ELISA kit (Cayman Chemical Company, Ann Arbor, MI, United States) following the manufacturer’s protocol. Standard 8-OHdG was assayed over a concentration range of 10.3–3.000 pg/mL in duplicate for each experiment.

### 2.4 Histochemical analysis

Sudan Black B (SBB) was used to confirm steatosis in the livers of HFD animals. Sections of livers were fixed in formol calcium, and 10-mm frozen sections were stained with SBB for fat detection according to standard procedures. All samples were imaged using a Leica microsystem (model DM6000B) and a Leica camera (model DFC 450 C).

### 2.5 Flow cytometry

To obtain a cell suspension from liver tissues, we used the Minute Cell Suspension Isolation Kit for Fresh/Frozen Tissues (Invent Biotechnologies, Plymouth, MN, United States). Briefly, 30 mg of liver tissue was homogenised in 200 μL of cold tissue dissociation buffer. The tissue was gently ground 200 times with a pestle and centrifuged at 500 × g for 3 min at 4 °C. The wet pellet was ground with the pestle for approximately 100–150 twists. Next, 1 mL of fluorescence-activated cell sorter (FACS) buffer was added and the sample was ground a few more times to resuspend. The cell suspension was passed through a cell strainer, and the resulting cells were collected by low-speed centrifugation (500 × g, 3 min). The Guava Autophagy LC3B Antibody-Based Assay Kit (Cytek Biosciences, Fremont, CA, United States) was used to assess autophagy in cell suspensions derived from fresh tissues. The cells were washed with ice-cold Phosphate buffer saline 1x (PBS1x), centrifuged, and then resuspended. Anti-LC3/FITC antibody was added to the cell suspension and incubated in the dark at room temperature for approximately 30 min. The cells were then washed once with 1X Assay Buffer to remove any residual or unbound antibody and centrifuged at 300 × g for 5 min. The pellet was resuspended in 200 µL of 1X Assay Buffer, and the data was acquired. Autophagy in the cells was assessed using the Guava EasyCyte 5 system (Cytek Biosciences, Fremont, CA, United States).

### 2.6 Preparation of total lysates from liver and cell

Tissue and cell lysates were prepared using RIPA buffer containing 150 mM sodium chloride (NaCl), 1.0% Triton X-100, 0.5% sodium deoxycholate, 0.1% sodium dodecyl sulfate (SDS), 50 mM Tris HCl (pH 8.0), 1 mM sodium orthovanadate (Na_3_VO_4)_, 1 mM phenylmethilsulfonyl fluoride (PMSF), and 1 mg/mL Leupeptin (Merck, Darmstadt, Germany). Subsequently, the homogenate was centrifuged at 16.000×g for 15 min at 4 °C (Beckman Coulter S.p.A., Milan, Italy). The protein concentrations of the supernatants of the centrifuged lysates were determined using BioRad’s DC method (Biorad Laboratories, Hercules, CA, United States).

### 2.7 Western blot analysis

Electrophoreses on Sodium dodecylsulfate polyacrylamide gel electrophoresis (SDS-PAGE) gels and Western blot (WB) analysis were performed essentially as described by Petito et al. (2023) and Senese et al. (2024) with minor modifications. Total lysates containing 30 μg protein for the liver and 15 μg protein for cell cultures were loaded in each lane and electrophoresed on SDS-PAGE gels and transferred to nitrocellulose membrane. The membranes were blocked with 5% nonfat dry milk in tris-buffered saline with 0.01% Tween 20 (TBS-T) (Merck, Darmstadt, Germany). Primary antibodies were diluted in TBS-T and 5% Bovine Serum Albumin (BSA) (Merck, Darmstadt, Germany), while secondary antibodies were diluted in TBS-T with 0.01% Tween 20% and 5% nonfat dry milk. Membranes were probed with the following antibodies: SREBP1c (1:1,000 dilution, Santa Cruz Biotechnology, sc-366, RRID:AB_2194229), p-PERK(Thr980) (1:1,000 dilution, Cell Signaling, Cat# 3179, RRID:AB_2095853), PERK (1:1,000 dilution, Cell Signaling, Cat# 3192, RRID:AB_2095847), p-eIF2α(Ser51) (1:1,000 dilution, Cell Signaling, Cat# 3398, RRID:AB_2096481), eiF2α (1:1,000 dilution, Cell Signaling, Cat#2103, RRID:AB_836874), ATF4 (1:1,000 dilution, Abclonal, Cat. A18687, RRID:AB_2862422), LC3B (1:1,000 dilution, Santa Cruz Biotechnology, sc-376404, RRID:AB_11150489), ATG5 (1:1,000 dilution, Novus Biologicals, NB110-53818, RRID:AB_828587), ATG16L1 (1:1,000 dilution, Novus Biologicals, NB110-82384, RRID:AB_1144849), ATG9A (1:1,000 dilution, Novus Biologicals, NB110-56893, RRID:AB_837629), Beclin-1 (1:1,000 dilution, Cell Signaling, Cat#3738, RRID:AB_490837), p62 (1:1,000 dilution, Cell Signaling, Cat#5114, RRID:AB_10624872), Caspase-3 (1:1,000 dilution, Cell Signaling, Cat#9662, RRID:AB_331439), Bcl-2 (1:1,000 dilution, Santa Cruz Biotechnology, sc-7382, RRID:AB_626736), B-ACTIN (1:1,000 dilution, Bioss Antibodies, bs-0061R, RRID:AB_10855480). As secondary antibodies, we used Goat Anti-Mouse IgG H&L (HRP) (Abcam Cat# ab97023, RRID:AB_10679675) and Goat polyclonal Secondary Antibody to Rabbit IgG - H&L (HRP) (Abcam Cat# ab97051, RRID:AB_10679369). Horseradish peroxidase (HRP)-conjugated secondary antibodies were used for signal detection by enhanced chemiluminescence using the Chemi Doc system and related software (Biorad Laboratories, Hercules, CA, United States). Antibody reference numbers and manufacturers are provided in Supplementary Table S1.

### 2.8 RNA purifcation

RNA purification was performed essentially as described by Senese et al. (2019). Two hundred microliters (200 µL) of serum was used to extract total RNA, including small RNAs (sRNA), using the miRNeasy Mini Kit (Qiagen, Hilden, Germany), following the supplementary protocol for miRNA isolation from serum. Total RNA, including sRNAs, was also extracted from liver tissues and cell culture pellets homogenized with a Polytron, using the miRNeasy Mini Kit (Qiagen, Hilden, Germany). RNA concentration was quantified spectrophotometrically using a NanoDrop 2000c (Thermo Scientific, Waltham, MA, United States).

### 2.9 miRNA profling

Reverse-transcription of RNA was performed by miScript II RT kit (Qiagen, Hilden, Germany), then aliquots of cDNA preparation were used for real-time polymerase chain reaction (RT-PCR) profling of mature miRNAs using Rat Serum&Plasma miRNA PCR Arrays (Qiagen, Hilden, Germany) in combination with miScript SYBR Green PCR kit (Qiagen, Hilden, Germany) and the MyiQ2 (Biorad Laboratories, Hercules, CA, United States) instrument. Data analysis was performed with the web-based sofware package for the miRNA PCR array system (http://pcrdataanalysis.sabiosciences.com/mirna/arrayanalysis.php). In brief, ΔCt value for each miRNA profiled in a plate is calculated using the formula ΔCt = CtmiRNA− Ctcel-miR-39. ΔΔCt for each miRNA across the two groups of samples (HFD and N animals) is calculated using the formula: ΔΔCt = ΔCt of HFD treated group − ΔCt of N group. Manufacturer’s technical notes recommend the use of a synthetic spike-in control for normalization when working with serum and plasma samples. The use of Cel-miR-39 as a normalizer for miRNA profiling on liquid biopsies is a generally accepted method (Madadi and Soleimani, 2019; Nomair et al., 2023; Roest et al., 2021; Senese et al., 2019; Stiuso et al., 2015; Wnuk et al., 2025). Expression fold-change was then obtained as 2^−ΔΔCt^, the normalized gene expression (2^−ΔCt^) in the HFD group divided the normalized gene expression (2^−ΔCt^) in the N group. Data are reported as fold-regulation, where fold-regulation is equal to the fold-change for fold-change values greater than one (upregulation) or is the negative inverse of the fold-change for fold-change values lower than one (downregulation) (Senese et al., 2019; Stiuso et al., 2015). The replicate 2^−ΔCt^ values for each miRNA in the N and HFD treatment groups were statistically analyzed by Student’s t-test and the criteria of differential expression were p < 0.05.

### 2.10 Quantitative PCR analysis

Total RNA (1 μg) was used to synthesize cDNA strands in a 20-μL-reaction volume using the SuperScript IV Reverse Transcriptase for RT-PCR (Thermo Scientific, Waltham, MA, United States). 50μM of random hexamers, 10 mM of dNTP mix, and 1 μg of total RNA were combined and heated at 65 °C for 5 min and then incubated on ice for at least 1 min. Annealed RNA was combined with reverse transcriptase (RT) reaction mix and incubated at 23 °C for 10min, 50 °C–55 °C for 10min, and 80 °C for 10 min. Real-Time quantitative RT-PCR (qRT-PCR) was conducted with 50 nM gene-specific primers, IQ SYBR Green supermix (Biorad Laboratories, Hercules, CA, United States), and cDNA samples (2 μL) in a total volume of 25 μL. A melting curve analysis was completed following amplification from 55 °C to 95 °C to ensure product identification and homogeneity. The mRNA expression levels were repeated in triplicate and were normalized to a reference gene (B-ACTIN and GAPDH, stable under specific experimental conditions) by using the 2^−ΔΔCT^ method. PCR primers were designed by using the Primer 3 program (Untergasser et al., 2012), and synthesized and verified by sequencing at Eurofins Genomics (Eurofins Genomics, Ebersberg, Germany). miR-18a-5p was quantified along with RNU6B (reference transcript) by qRT-PCR with TaqMan® miRNA assays from Applied Biosystems, in conformity with the manufacturer’s protocol. The expression levels were normalized to a reference gene (RNU6B) by using the 2^−ΔΔCT^ method. The analyses were performed on six independent experiments for animal model and three independent experiments for cell culture model, each in triplicate. Primer are listened in Tables 1, 2.

**TABLE 1:** Forward and reverse primers (rat) used in qRT-PCR.

Gene name (Rat)	Forward primer (5′→3′)	Reverse primer (5′→3′)
Srebp1c	CTGTCGTCTACCATAAGCTGCAC	ATAGCATCTCCTGCACACTCAGC
Perk	GAAGTGGCAAGAGAGATGG	GAGTGGCCAGTCTGTGCTTT
Eif2α	GCAAACAATGTCCCATCCTT	CCCACACTTCACAGAGAGCA
Dgat1	GGGAACCCACTGGAGTGATA	ACCTGGCCACAATTATCTGC
Agpat1	TATTTGACGTGGAGCAGCAG	CCTCTTCCTGGCAATACTCG
Gapdh	GCACCGTCAAGGCTGAGAAC	TGGTGAAGACGCCAGTGGA
β-actin	GGAGATTACTGCCCTGGCTCCTA	GACTCATGGTACTCCTGCTTCCTG

**TABLE 2:** Forward and reverse primers (Human) used in qRT-PCR.

Gene name (Human)	Forward primer (5′→3′)	Reverse primer (5′→3′)
Srebp1c	ACACAGCAACCAGAAACTCAAG	AGTGTGTCCTCGACCTCAGTC
Perk	TGTCGCCAATGGGATAGTGACGAA	AATCCGGCTCTCGTTTCCATGTCT
Eif2α	CGTTGCCCAGGACAGTATTT	GGGACTACTGCACTCCTTCG
β-actin	TGCTCCTCCTGAGCGCAAGTA	CCACATCTGCTGGAAGGTGGA
Gapdh	GGAGCGAGATCCCTCCAAAAT	GGCTGTTGTCATACTTCTCATGG

### 2.11 Cell culture

In this study, HepG2 cells were cultured in Dulbecco’s Modified Eagle’s Medium (DMEM) (Thermo Scientific, Waltham, MA, United States) supplemented with 100 U/mL penicillin, 100 U/mL streptomycin, and 10% Fetal Bovine Serum (FBS) (Thermo Scientific, Waltham, MA, United States) at 37 °C in a humidified atmosphere of 5% CO_2_. All cells were plated in a 6-well plate at least 48 h before treatment. At 80% confluence, HepG2 cells were exposed to a mixture of long-chain Free Fatty Acids (FFAs), oleate and palmitate (1.5 mM final concentration, 2:1 M ratio) (Merck, Darmstadt, Germany), for 24 h in media containing 1% BSA free fatty acids (Grasselli et al., 2011). For the same period of time, control hepatocytes were incubated without FFAs. To achieve the desired final concentrations, stock solutions of 30 mM FFAs were diluted in culture medium containing 1% BSA free fatty acids. At the end of the treatment, cells were scraped, washed twice with PBS1x, and stored at −80 °C until further use. The post stimulation medium was cleared of cell debris by centrifugation at 500 × g for 5 min at room temperature, transferred to a new tube, and stored at −80 °C. For each analysis, experiments were performed with three biological replicates.

### 2.12 Cell culture transfections

HepG2 cells were cultured in DMEM supplemented with 100 U/mL penicillin, 100 U/mL streptomycin, and 10% FBS at 37 °C in a humidified atmosphere of 5% CO_2_. Before the transfection, cells were trypsinized and seeded in 6-well plates. Prior to transfection, cells were incubated for 24–48 h until confluence reached 80%–90%. For transfection, 3 µL of Lipofectamine 3000 (Thermo Scientific, Waltham, MA, United States) was mixed with 10 µM hsa miR-18a-5p mimic or 10 µM miRNA mimic Negative Control (both from Thermo Scientific, Waltham, MA, United States) in serum and antibiotic-free medium. After incubating the mixture at room temperature for 20 min, it was added to each well, and cells were cultured in a humidified atmosphere with 5% CO_2_ at 37 °C. After 36 h, the transfection mix was replaced with complete medium containing a mixture of oleate and palmitate (1.5 mM final concentration, 2:1 M ratio) for an additional 24 h. At the end of the treatment, cells were scraped, washed twice with PBS1x, and stored at −80 °C until further use. For each analysis, experiments were performed with three biological replicates.

### 2.13 Oil Red O (ORO) staining

To determine triglyceride accumulation in HepG2 cells, ORO staining was performed using 6-well culture plates. After 24 h of incubation with the FFA mixture, cells were washed twice with ice-cold PBS 1x and fixed with 10% formalin for 10 min. The formalin was then discarded, and the cells were exposed to 10% formalin for an additional 1 h. After fixation, cells were washed four times with distilled water and then rinsed with 60% isopropanol for 5 min. Cells were stained with ORO working solution for 10 min at room temperature and washed again with PBS1x to remove unbound stain. Images were acquired under an optical microscope at 10X and 40X magnifications. To quantify the ORO content, 60% isopropanol was added to each sample. After shaking at room temperature for 5 min, the absorbance of the samples was measured at 500 nm using a BioTek Synergy H1 spectrophotometer (Agilent Technologies, Santa Clara, CA, United States).

### 2.14 MTT assay

HepG2 cells were plated (100 μL/well) in triplicate in 96-multiwell plates at a density of 1.5 × 10^4^ cells/well. The day after seeding, cells were incubated with a mixture of oleate and palmitate (1.5 mM final concentration, 2:1 M ratio) for 24 h. After 24 h of incubation, cells were treated with 150 µL of 0.5 mg/mL 3-(4,5-dimethyl-2-thiazolyl)-2,5-diphenyl-2H-tetrazolium (MTT) (Merck, Darmstadt, Germany), having previously been dissolved in FBS-free culture medium for 4 h at 37 °C in a 5% CO_2_ humidified atmosphere. The MTT solution was then removed and 100 µL of dimethyl sulfoxide (DMSO) (Merck, Darmstadt, Germany) was added to dissolve the produced formazan dye. Finally, the absorbance at 570 nm of each sample was determined using a BioTek Synergy H1 spectrophotometer (Agilent Technologies, Santa Clara, CA, United States).

### 2.15 Immunofluorescence (IF) staining

The IF staining was performed according to our previous paper (Venditti et al., 2020). Briefly, cells were fixed in 4% paraformaldehyde (Sigma-Aldrich, St. Louis, MO, United States) solution for 15 min after the treatment at room temperature (20 °C–23 °C), and then permeabilized with 0.3% Triton-X100 for 10 min. Later, nonspecific binding sites were blocked with a blocking solution (5% normal goat serum, 0.1% Triton-X100 in PBS1x) for 1 h at room temperature before the addition of anti-LC3 and anti-Caspase-3 antibodies, both diluted 1:100, for overnight incubation at 4 °C. After three washes in PBS1x, slides were incubated for 1 h with the appropriate secondary antibody (#A32731; Alexa Fluor 488, Thermo Fisher Scientific, Waltham, Ma, United States; SAB4600082; Anti-Mouse IgG, Sigma–Aldrich, St. Louis, MO, United States) diluted 1:500. Slides were washed again three times in PBS1x, and cells nuclei were stained with 4′,6-diamidino-2-phenylindole (DAPI) mounting medium (Abcam, #ab104139, Cambridge, United Kingdom). Photomicrographs were taken with a fluorescence microscope (Leica DM 5000 B + CTR 5000; Leica Microsystems, Wetzlar, Germany). Densitometric analysis of immunofluorescence signals and were performed with Fiji plugin (version 3.9.0/1.53 t) of ImageJ Software by counting at least 300 cells in different microscopic fields.

### 2.16 Statistical analysis

All results were analyzed with the GraphPad Prism 8.0.1 software system (GraphPad Software, San Diego, CA, United States). Data were expressed as the mean ± SEM and were normally distributed. The statistical significance of the differences between experimental groups was determined using one way ANOVA followed by Tukey *post hoc* testing and Student’s t-test for between-group comparisons. Differences were considered statistically significant at p < 0.05.

## 3 Results

### 3.1 The HFD for 5 weeks induces MASLD in rats

Metabolic parameters were determined in rats fed a standard diet (N) and in rats fed a HFD. As expected, HFD-fed animals gained significantly more weight compared to those on the standard diet. This was associated with an increase in food intake in the HFD group compared to animals on the normal diet. The liver-to-body weight (LW/BW) ratio was 3.38 ± 0.19 in the HFD rats, compared to 2.89 ± 0.28 in the N group (not significant). HFD-fed rats also showed significantly higher serum levels of cholesterol and triglycerides. Serum Alanine Aminotransferase (ALT) levels were increased, suggesting hepatocyte damage. Moreover, an increase in serum levels of 8-OHdG, an oxidized derivative of the guanine base considered a marker of systemic oxidative stress, was also observed (Table 3).

**TABLE 3:** Body Weight gain (BW), Food intake, LW/BW, serum Cholesterol, serum Triglycerides, serum ALT and serum 8-OHdG levels in N and HFD rats.

Parameters	N	HFD
BW (g)	377.0 ± 9.2	450.0 ± 6.1*
Food intake (g)	37.9 ± 0.9	42.2 ± 1.1*
LW/BW (%)	2.9 ± 0.3	3.38 ± 0.2
Cholesterol (mg/dL)	53.0 ± 2.3	88.0 ± 3.4*
Triglycerides (mg/dL)	121.0 ± 8.1	255.0 ± 10.6*
ALT (U/l)	24 ± 1.4	30 ± 2.2*
8-OHdG (pg/mL)	762.9 ± 50.5	965.9 ± 142.6

Body weight gain (g), Food intake (g), LW/BW (%), serum Cholesterol (mg/dL), serum Triglycerides (mg/dL), serum ALT (U/l), serum 8-OHdG (pg/mL) in N and HFD rats. N: rats receiving a standard diet for 5 weeks; HFD: rats fed on a high-fat diet for 5 weeks. Values are the means ± SEM of five different rats (n = 6). Values with different symbols are significantly different: *P < 0.05 vs. N.

**FIGURE 1:**
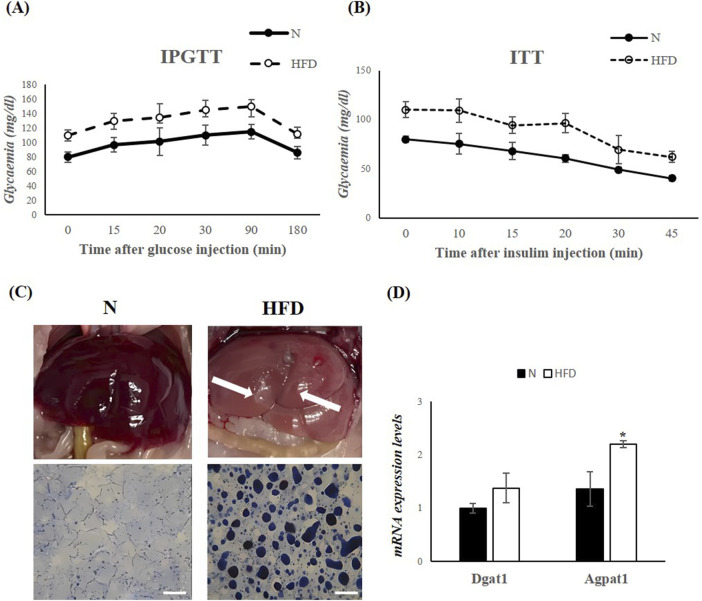
HFD for 5 weeks leads to MASLD development in Rats. **(A,B)** IPGTT and ITT in N and HFD rats. Blood glucose levels are expressed as mg/dL. **(C)** Morphological differences in the liver of N and HFD rats. The fatty liver has a yellowish appearance (arrows highlighting liver colors). Hepatic fat accumulation (assessed by SBB) in N and HFD rats, Scale bars: 100 μm **(D)** RT-qPCR analysis of Dgat1 and Agpat1 in liver samples of N and HFD rats. N: rats receiving a standard diet for 5 weeks; HFD: rats fed on a high-fat diet for 5 weeks. Values are the means ± SEM of six different rats (n = 6). Values with different symbols are significantly different: *P < 0.05 vs. N.

The Intraperitoneal Glucose Tolerance Test (IGPTT) revealed that, following a glucose load, HFD-fed rats exhibited impaired glucose tolerance compared to N rats (Figure 1A). In addition, the Insulin Tolerance Test (ITT) showed that the reduction in blood glucose levels after insulin administration was compromised in HFD animals (Figure 1B). These results suggest that the HFD induces insulin resistance and contributes to the development of metabolic dysfunction. Morphological differences were observed in the livers of N and HFD rats (Figure 1C). As a result of lipid accumulation, the liver of HFD-fed animals appeared pale in color (Figure 1C). This observation was further supported by SBB staining, which confirmed significantly increased hepatic lipid accumulation in the HFD group compared to controls (Figure 1C). Furthermore, the expression levels of 1-Acyl-snGlycerol-3-Phosphate Acyltransferase Alpha (Agpat1) and Diacylglycerol O-Acyltransferase 1 (Dgat1), two genes involved in triglyceride synthesis (Takeuchi and Reue, 2009), were increased in the liver of HFD rats, highlighting the increase in triglyceride synthesis in animal fed an HFD (Figure 1D). All the above results demonstrate that the HFD-fed rat model shows all the key characteristics of MASLD.

### 3.2 The HFD induces a down-regulation of miR-18a-5p with an up-regulation of SREBP1c and of key components of the UPR^ER^ signaling pathways

**FIGURE 2:**
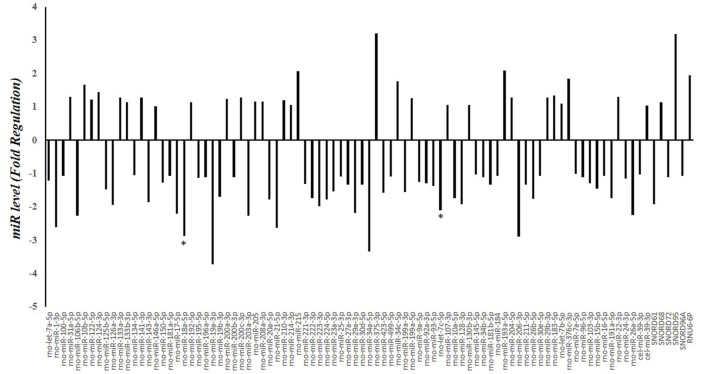
Serum miRNA Profling in N and HFD Rats. Fold regulation of miRNA levels detectable in the serum of 6 HFD rats versus 6 N rats (n = 6). N: rats receiving a standard diet for 5 weeks; HFD: rats fed on a high-fat diet for 5 weeks. Values with different symbols are significantly different: *P < 0.05 vs. N.

A screening of circulating miRNAs revealed both upregulation and downregulation of several miRNAs in the serum of HFD-fed rats compared to N rats. In particular, miR-18a-5p and let-7c-5p were significantly downregulated by 2.87 and 2.1 fold, respectively (Figure 2).

**FIGURE 3:**
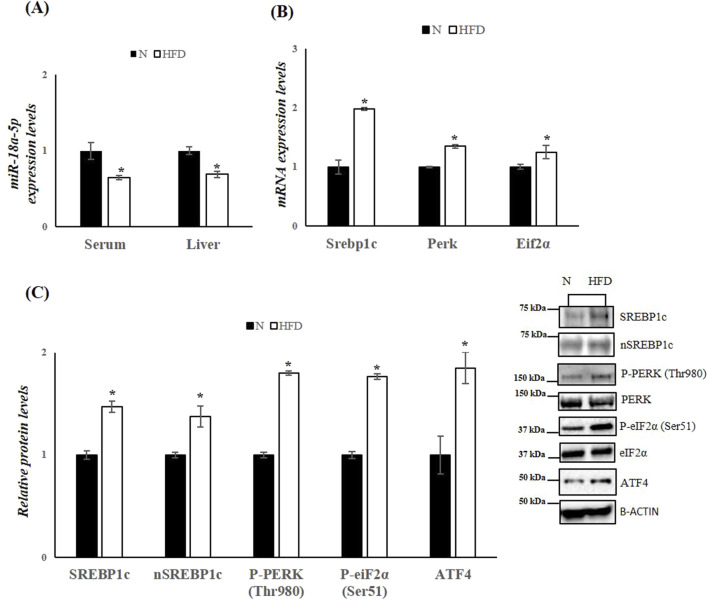
HFD affects miR-18a-5p expression and activates the UPR^ER^ pathway in the liver of rats. **(A)** qRT-PCR analysis of miR-18a-5p in serum and liver samples of N and HFD rats **(B)** qRT-PCR analysis of Srebp1c, Perk and Eif2α in liver samples of N and HFD rats **(C)** WB images and densitometry of SREBP1c, nSREBP1c, P-PERK (Thr980), P-eiF2α(Ser51) and ATF4 in liver samples of N and HFD rats. Histograms show the results of densiometric analysis of immunoblots. The protein level was normalized to that of B-ACTIN and/or to the total forms for phosphorylated proteins. Representative blots are shown. N: rats receiving a standard diet for 5 weeks; HFD: rats fed on a high-fat diet for 5 weeks. Values are the means ± SEM of six different rats (n = 6). Values with different symbols are significantly different: *P < 0.05 vs. N.

miR-18a-5p was further validated (Figure 3). The data confirm an approximately 36% decrease in serum miR-18a-5p levels and a 31% decrease in liver miR-18a-5p expression in HFD rats compared to N rats (Figure 3A). Based on the prediction of a conserved miR-18a-5p binding site on 3′UTR of SREBP1c, we measured the hepatic mRNA and protein levels of the SREBP1c. As expected, hepatic mRNA and protein levels of SREBP1c were significantly increased in HFD-fed rat when compared to those on the standard diet (Figures 3B,C). Given that the active nuclear form of SREBP1 can bind to the PERK promoter (Hu et al., 2020), thereby enhancing its expression, and considering that the initial ER stress response involves phosphorylation of eIF2α and increases in Activating transcription factor 4 (ATF4) protein levels, we next examine these markers. The results showed significantly higher expression levels of Perk and eIF2α in liver samples from HFD animals (Figure 3B). Additionally, phosphorylation levels of these markers, as well as ATF4 protein levels, were significantly enhanced in the liver of HFD animals compared to the N group, indicating ER stress and activation of the UPR induced by FAs (Figure 3C).

### 3.3 The HFD affects autophagy and apoptosis in response to ER stress in rats

**FIGURE 4:**
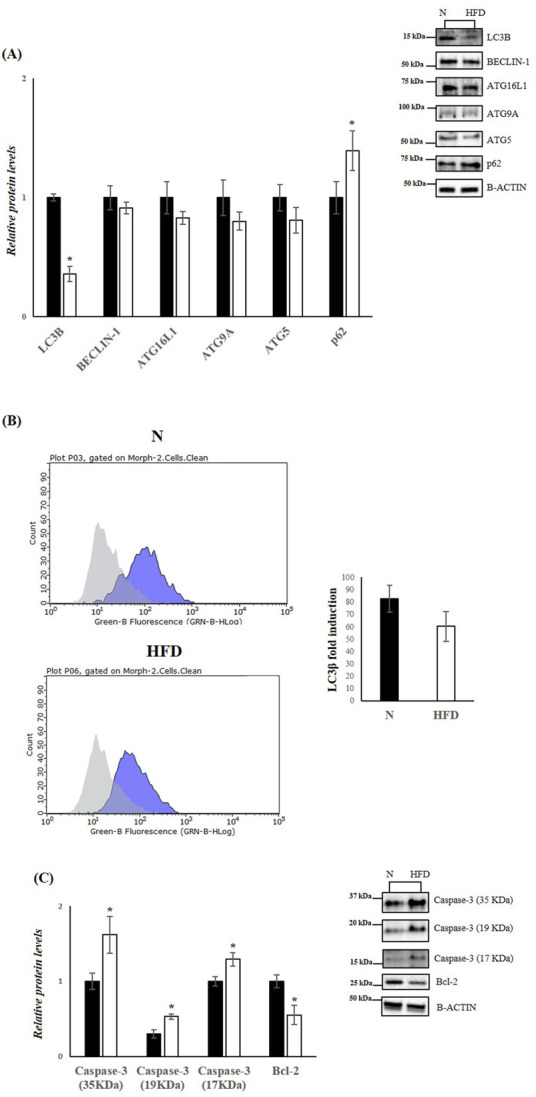
HFD affects Autophagy and Apoptosis in the liver of rats. **(A,C)** WB images and densitometry of LC3B, BECLIN-1, ATG16L1, ATG9A, ATG5, p62, Caspase-3 (35, 19 and 17 kDa) and Bcl-2 in liver samples of N and HFD rats. Histograms show the results of densiometric analysis of immunoblots. The protein level was normalized to that of B-ACTIN. Representative blots are shown. **(B)** Representative figures of flow cytometry analysis in liver samples of N and HFD rats. ΔMFI (delta mean fluorescence intensity), is calculated from the MFI of the cells expressing the marker of interest divided by the MFI of the cells stained with the isotype control, and these numbers are shown in the histogram. N: rats receiving a standard diet for 5 weeks; HFD: rats fed on a high-fat diet for 5 weeks. Values are the means ± SEM of six different rats (n = 6). Values with different symbols are significantly different: *p < 0.05 vs. N.

In obesity and MASLD, the UPR^ER^ and autophagy pathways interact dynamically. In obese mice, impaired hepatic autophagy leads to ER stress, and restoring autophagy can improve ER homeostasis (Yang et al., 2010). Our analysis revealed that in the livers of HFD rats, the levels of key autophagic proteins such as Microtubule-associated protein 1A/1B light chain 3B (LC3B), Beclin-1, Autophagy-related 16-like 1 (ATG16L1), Autophagy-related 5 (ATG5), and Autophagy-related 9A (ATG9A) were reduced (Figure 4A). Conversely, the protein levels of sequestosome 1 (SQSTM1 (p62)), which inhibits autophagy when elevated, were significantly increased in HFD rats compared to the N group. The decrease in autophagy was further confirmed by cytofluorimetric analysis, which detected reduced levels of LC3B in cells isolated from the livers of HFD rats compared to the control group (Figure 4B), Given the central role of PERK in UPR^ER^ signalling, it is likely that this UPR mediator is also crucial for ER stress-induced apoptosis. Prolonged ER stress leads to the accumulation of misfolded proteins, which in turn promotes apoptotic pathways (Henkel and Green, 2013). To explore this, we measured hepatic protein levels of Caspase-3 (35, 19, and 17 kDa), a key mediator of programmed cell death, and B-cell lymphoma 2 (Bcl-2), an anti-apoptotic factor. Western blot analysis revealed a significant downregulation of Bcl-2 and a significant upregulation of Caspase-3, indicating enhanced apoptotic processes in the livers of HDF rats (Figure 4C).

### 3.4 FAs treatment induce down-regulation of miR-18a-5p with modulation of SREBP1c and UPR^ER^ markers in HepG2 cells

**FIGURE 5:**
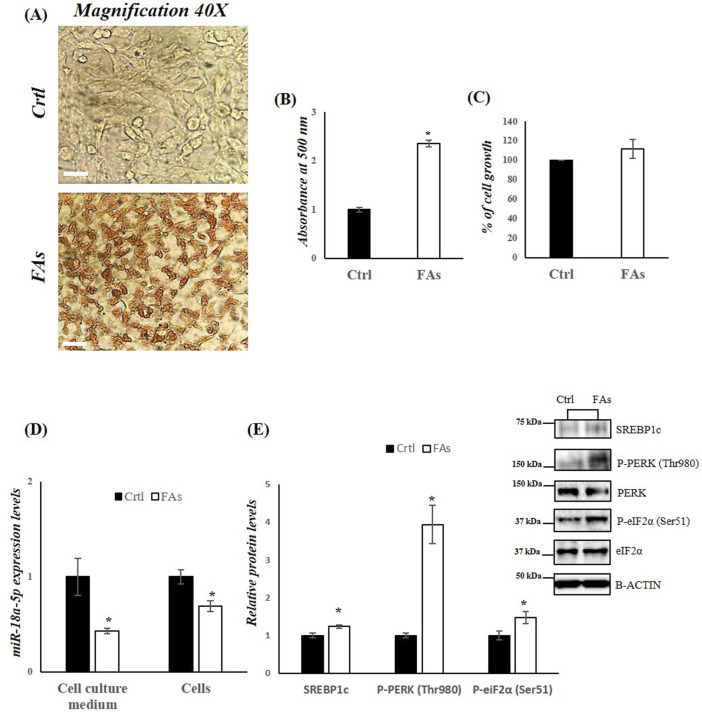
FAs mixture affects miR-18a-5p expression and activates the UPR^ER^ pathway in HepG2 cells. **(A)** ORO staining of TG accumulation in HepG2 cells treated with or without FAs. Original magnification: 40x. Scale bar = 100 μm **(B)** Oil Red O quantification in HepG2 cells treated with or without FAs **(C)** MTT assay to measure cell viability in HepG2 cells treated with or without FAs **(D)** qRT-PCR analysis of miR-18a-5p in cell culture medium and HepG2 cells treated with or without FAs **(E)** WB images and densitometry of SREBP1c, P-PERK (Thr980) and P-eiF2α (Ser51) in HepG2 cells treated with or without FAs. Histograms show the results of densiometric analysis of immunoblots. The protein level was normalized to that of B-ACTIN and/or to the total forms for phosphorylated proteins. Representative blots are shown. Ctrl: HepG2 cells treated with 1% BSA-free fatty acids for 24 h; FAs: HepG2 cells treated with oleate and palmitate (1.5 mM final concentration, 2:1 M ratio) for 24 h. Values are the means ± SEM of three independent experiments (n = 3). Values with different symbols are significantly different: *p < 0.05 vs. Ctrl.

To further investigate the role of miR-18a-5p in hepatic lipid accumulation, we used an *in vitro* model of MASLD. HepG2 cells were treated with FAs mixture for 24 h to induce MASLD. The results revealed differences in lipid accumulation in HepG2 cells treated with FAs when compared to the control group (Figures 5A,B). Lipid droplets in the treated cells were stained red, indicating significant lipid accumulation (Figures 5A,B). To assess whether the fatty acid mixture was cytotoxic, we performed MTT assays on HepG2 cells. Our results indicated that lipid mixture did not affect cell viability (Figure 5C).

In HepG2 cells treated with FAs, as well as in the cell culture medium isolated from these cell, we observed a significant reduction in miR-18a-5p expression levels compared to control group (Ctrl) (Figure 5D). Consequently, a significant increase in the protein levels of SREBP1c as well as in the phosphorylation levels of PERK and eIF2α, was observed in the FA-treated group compared to the Ctrl group (Figure 5E).

### 3.5 FAs treatment affects autophagy and apoptosis in HepG2 cells

**FIGURE 6:**
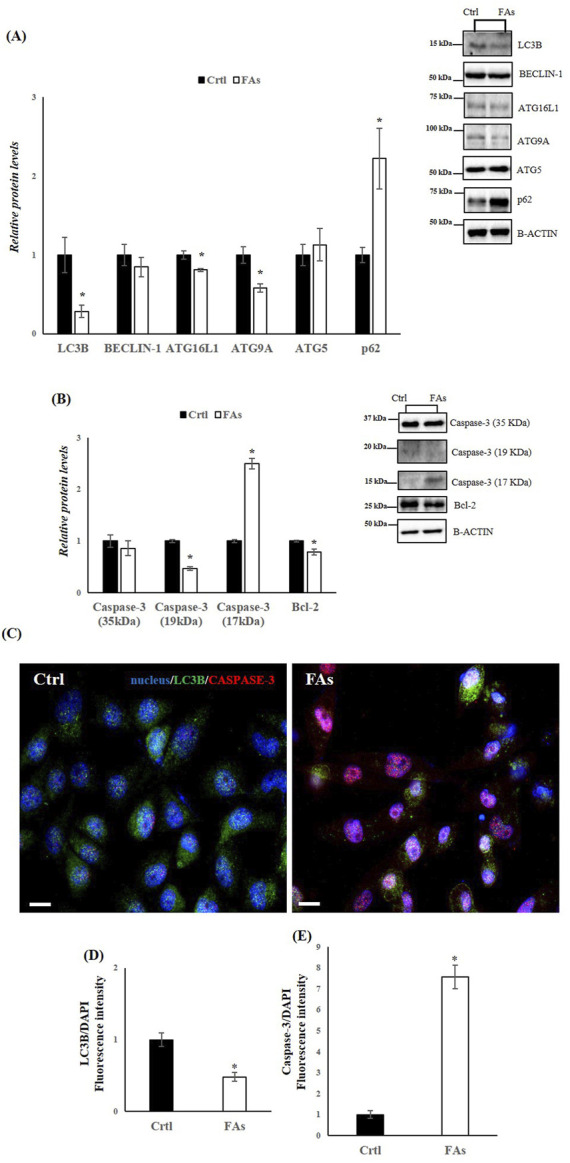
FAs mixture affects Autophagy and Apoptosis in HepG2 cells. **(A,B)** WB images and densitometry of LC3B, BECLIN-1, ATG16L1, ATG9A, ATG5, p62, Caspase-3 (35, 19 and 17 kDa), and Bcl-2 in HepG2 cells treated with or without FAs. Histograms show the results of densiometric analysis of immunoblots. The protein level was normalized to that of B-ACTIN. Representative blots are shown **(C)** IF analysis of LC3B and Caspase-3 in HepG2 cells treated with or without FAs. Slides were counterstained with DAPI (blue), which mark the nucleus. Original Magnification 40x, scale bars 20 μm **(D)** and **(E)** Histogram showing the quantification of LC3B and Caspase-3 fluorescence signal intensity with respect to DAPI signal. Crtl: HepG2 cells treated with 1% BSA-free fatty acids for 24 h; FAs: HepG2 cells treated with oleate and palmitate (1.5 mM final concentration, 2:1 M ratio) for 24 h. Values are the means ± SEM of three independent experiments (n = 3). Values with different symbols are significantly different: *p < 0.05 vs. Crtl.

In the *in vitro* model, we also assessed the levels of key proteins involved in autophagy and apoptosis. WB analysis revealed a downregulation of LC3B, Beclin-1, ATG16L1, and ATG9A in the FA-treated group compared to the Ctrl group (Figure 6A). In contrast, the protein levels of SQSTM1 (p62), which inhibits autophagy when elevated, were significantly increased in cells exposed to FAs (Figure 6A). Furthermore, the protein levels of Caspase-3 (17 kDa) were significantly elevated, while Bcl-2 levels were significantly reduced in the FA-treated group (Figure 6B). To further characterize the effects of FAs on autophagy and apoptosis, we performed IF analysis on HepG2 cells (Figures 6C,D). Compared to the FA group, the Ctrl group showed a distinct immunosignal for LC3B (green), indicating normal autophagic activity (Figures 6C,D). In contrast, the FA group exhibited a stronger immunosignal for Caspase-3 (red), suggesting an increase in apoptotic activity compared to the Ctrl group (Figures 6C–E). These results collectively suggest impaired autophagic flux and increased apoptosis in the FA-treated HepG2 cells.

### 3.6 The overexpression of miR-18a-5p reduces fat accumulation and ER stress in HepG2 cells treated with FAs

**FIGURE 7:**
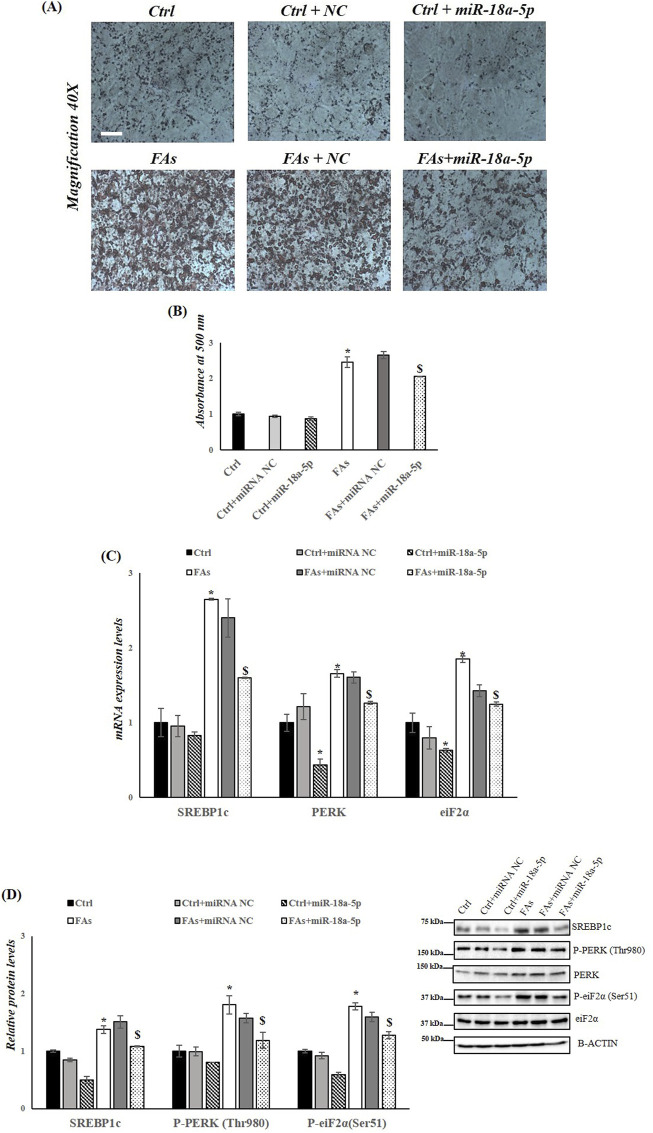
miR-18a-5p Overexpression reduces fat accumulation and ER Stress in HepG2 cells. **(A)** ORO staining of triglycerides accumulation in HepG2 cells transfected with or without miR-18a-5p. Original magnification: 40x. Scale bar = 100 μm **(B)** ORO quantification in HepG2 cells transfected with or without miR-18a-5p **(C)** qRT-PCR analysis of Srebp1c, Perk, and Eif2α in HepG2 cells transfected with or without miR-18a-5p **(D)** WB images and densitometry of SREBP1c, P-PERK (Thr980), and P-eiF2α (Ser51) in HepG2 cells transfected with or without miR-18a-5p. Histograms show the results of densiometric analysis of immunoblots. The protein level was normalized to that of B-ACTIN and/or to the total forms for phosphorylated proteins. Representative blots are shown. Crtl: HepG2 cells treated with 1% BSA-free fatty acids for 24 h; Ctrl + miRNA NC: HepG2 cells transfected with miRNA mimic Negative Control for 36 h; Ctrl + miR-18a-5p: HepG2 cells transfected with miR-18a-5p for 36 h; FAs: HepG2 cells treated with oleate and palmitate (1.5 mM final concentration, 2:1 M ratio) for 24 h; FAs+ miRNA NC: HepG2 cells transfected with miRNA mimic Negative Control for 36 h; FAs + miR-18a-5p: HepG2 cells transfected with miR-18a-5p for 36 h and subsequently treated with oleate and palmitate (1.5 mM final concentration, 2:1 M ratio) for 24 h. Values are the means ± SEM of three independent experiments (n = 3). Values with different symbols are significantly different: *P < 0.05 vs. Crtl; $P < 0.05 vs. FAs and Ctrl + miR-18a-5p.

To further confirm the role of miR-18a-5p in the MASLD, HepG2 cells were transfected with miR-18a-5p mimic and subsequently exposed to a FAs mixture. Our results showed that overexpression of miR-18a-5p reduced fat accumulation in HepG2 cells treated with FAs (FAs + miR-18a-5p mimic) compared to the FAs group (Figures 7A,B). As expected, the expression levels of SREBP1c, PERK, and eIF2α were significantly reduced in Ctrl+ miR-18a-5p mimic and FAs+ miR-18a-5p mimic groups compared to their respective controls (Ctrl and FAs) (Figure 7C). In addition, protein levels of SREBP1c and phosphorylation levels of PERK and eIF2α were significantly decreased in Ctrl + miR-18a-5p mimic and FAs + miR-18a-5p mimic when compared to Ctrl and FAs groups, respectively (Figure 7D).

### 3.7 The overexpression of miR-18a-5p improves the autophagic flux and reduces the apoptotic activity in HepG2 cells treated with FAs

**FIGURE 8:**
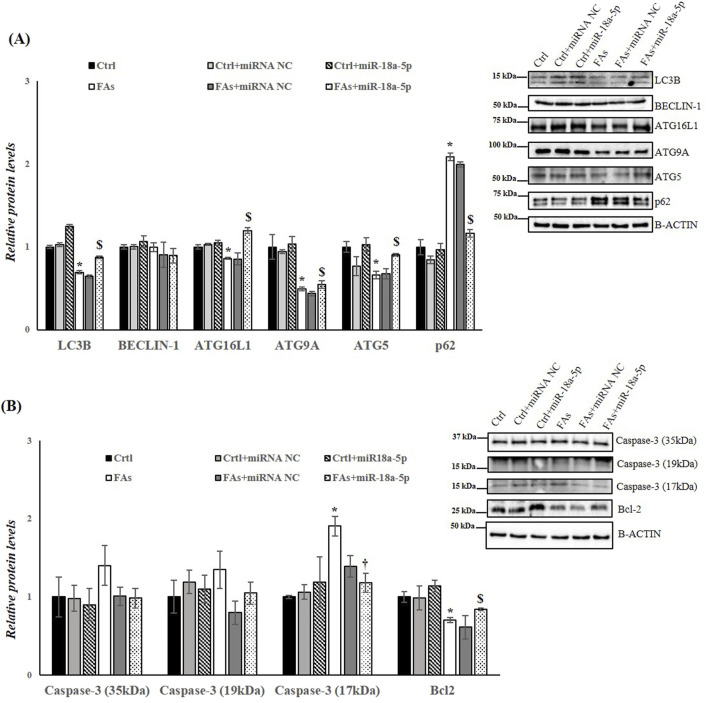
miR-18a-5p Overexpression modulates Autophagy and Apoptosis in HepG2 Cells. **(A,B)** WB images and densitometry of LC3B, BECLIN-1, ATG16L1, ATG9A, ATG5, p62, Caspase-3 (35, 19 and 17 kDa), and Bcl-2 in HepG2 cells transfected with or without miR-18a-5p. Histograms show the results of densiometric analysis of immunoblots. The protein level was normalized to that of B-ACTIN and/or to the total forms for phosphorylated proteins. Representative blots are shown. Ctrl: HepG2 cells treated with 1% BSA-free fatty acids for 24 h; Crtl + miRNA NC: HepG2 cells transfected with miRNA mimic Negative Control for 36 h; Ctrl + miR-18a-5p: HepG2 cells transfected with miR-18a-5p for 36 h; FAs: HepG2 cells treated with oleate and palmitate (1.5 mM final concentration, 2:1 M ratio) for 24 h; FAs+ miRNA NC: HepG2 cells transfected with miRNA mimic Negative Control for 36 h; FAs + miR-18a-5p: HepG2 cells transfected with miR-18a-5p for 36 h and subsequently treated with oleate and palmitate (1.5 mM final concentration, 2:1 M ratio) for 24 h. Values are the means ± SEM of three independent experiments (n = 3). Values with different symbols are significantly different: *P < 0.05 vs. Ctrl; $P < 0.05 vs. FAs and Ctrl + miR-18a-5p.

Finally, to assess the involvement of miR-18a-5p in autophagy flux and in apoptosis in response to FAs-induced ER stress, we analyzed the protein levels of several markers of autophagy and apoptosis. The results highlight an increase in autophagy flux in cells overexpressing miR-18a-5p and treated with FAs when compared to the cells treated only with FAs. Specifically, in HepG2 cells transfected with miR-18a-5p mimic and treated with FAs, we observed a significant increase in protein levels of LC3B, ATG16L1, ATG9A and ATG5, as well as a significant decrease in protein levels of p62 (Figure 8A). Furthermore, we confirmed the involvement of miR-18a-5p in apoptosis *in vitro*. Our findings showed that the overexpression of the miR-18a-5p is associated with a reduction in Caspase-3 levels and an increase in the Bcl-2 protein levels, ultimately reducing the process of programmed cell death (Figure 8B).

## 4 Discussion

The global prevalence of MASLD has risen dramatically over the past two decades, paralleling the surge in obesity rates. This complex disease requires a deep understanding of the molecular mechanisms underlying its pathogenesis. Among molecular mechanisms implicated in MASLD development, the interplay between lipid metabolism and inflammation has emerged particularly important. It has been proposed that fatty acids, particularly saturated fatty acids may act as endogeneous ligands of Toll-like receptors 4 (TLR4), thereby promoting inflammation and dylipidemia-associated liver disease (Erridge and Samani, 2009). Therefore, inflammatory processes may play key roles in the progression and pathogenesis of fatty liver diseases. In addition, the spleen–liver axis contributes to MASLD pathogenesis by facilitating immune cell recruitment and amplifying inflammatory cascades. Fatty liver inflammation is associated with an enrichment of splenic myeloid-derived suppressor cells (MDSCs) and natural killer T (NKT) cells in the liver, suggesting the involvement of a cell-specific axis (Brummer et al., 2025; Tarantino et al., 2021). Moreover, leukotriene B_4_ (LTB_4_), released from the spleen, acts as an endocrine mediator that enhances hepatic production of pro-inflammatory cytokines, including Tumor Necrosis Factor (TNF), during systemic inflammation (Fonseca et al., 2021).

MASLD is also characterized by a profound reprogramming of hepatic metabolism, with epigenetic mechanisms, including the dysregulation of miRNA expression, playing a pivotal role in the onset of steatosis and its progression to more severe disease stages.

This study highlights the role of miR-18a-5p in modulating lipid metabolism, ER stress, and liver cell survival within the context of MASLD induced by a HFD. Our findings indicate that miR-18a-5p modulates key pathways involved in disease pathogenesis, including the SREBP1c-dependent lipogenesis pathway and the UPR. Together, these pathways contribute to hepatic lipid accumulation and dysfunction.

In agreement with previous studies, the HFD animal model used in this study successfully replicated several metabolic and histological hallmarks of MASLD. Rats fed a HFD for 5 weeks exhibited significantly greater weight gain compared to controls on a standard diet, reflecting increased adiposity due to dietary fat intake (Senese et al., 2019). Morphological analysis confirmed hallmark features of lipid accumulation, including pale liver coloration and intensified SBB staining, increased lipid content.

Elevated serum levels of cholesterol and triglycerides confirmed the presence of a dyslipidemic phenotype associated with HFD-induced obesity. In parallel, upregulation of genes involved in triglyceride synthesis, including Agpat1 and Dgat1 further highlighted the role of HFDs in promoting lipogenesis, an essential contributor to MASLD progression (Ferramosca and Zara, 2014). Increased serum ALT levels indicate hepatocyte damage, while elevated levels of 8-OHdG, a marker of oxidative stress, reflected the oxidative burden associated with excessive fat intake (Petito et al., 2021; Pilger and Rüdiger, 2006). Oxidative stress play a key role in MASLD pathogenesis, driving lipid peroxidation, inflammation, and hepatocyte injury (Tanaka et al., 2008). Impaired IGPTT and ITT results confirmed the presence of insulin resistance, a well-established and central mechanism in MASLD development (Maldonado-Rojas et al., 2024).

miRNAs are critical regulators of MASLD and metabolic dysfunction (Gjorgjieva et al., 2019; Ha and Kim, 2014). Several miRNAs modulate genes expression involved in hepatic lipid and cholesterol metabolism, making them attractive therapeutic targets. Our miRNA profiling revealed significant alterations in circulating miRNAs in HFD-fed animals, with miR-18a-5p showing the most pronounced downregulation. Further validation confirmed reduced miR-18a-5p expression in both serum and liver tissue after 5 weeks of HFD feeding. In addition to miR-18a-5p, we also observed a significant reduction of let-7c-5p levels in the serum of HFD-fed rats compared to those fed a normal diet. The expression of this miRNA was found to be reduced in both *in vivo* and *in vitro* models of ethanol-induced hepatic steatosis, suggesting a potential involvement of miR-let-7c-5p in liver disease (Wang et al., 2018).

SREBP1c, a master transcriptional regulator of lipogenesis, is activated in ER membrane through proteolytic cleavage facilitated by sterol-regulatory element-binding protein cleavage-activating protein (SCAP). Its active nuclear form (nSREBP) translocates to the nucleus, where it modulates the expression of lipogenic genes (Bennett et al., 2004; Bertolio et al., 2019). In our study, HFD-fed rats exhibited increased hepatic mRNA and protein levels of SREBP1c, including its active nuclear form. Bioinformatic analysis predicted a conserved miR-18a-5p binding site in the 3′ UTR of SREBP1c (Zhang et al., 2019), supporting our hypothesis that downregulation of miR-18a-5p contributes to increased hepatic SREBP1c expression and, consequently, enhanced lipogenesis and hepatic steatosis (Ju et al., 2020; Wang et al., 2015; Zhao et al., 2018). These data position miR-18a-5p as a potential post-transcriptional regulator of hepatic metabolism and a promising therapeutic target in MASLD.

Excessive FAs from HFDs induce chronic ER stress, thereby impairing protein folding and lipid metabolism. The UPR, an adaptive response that aims to restore ER homeostasis, is activated through three primary pathways: inositol-requiring enzyme 1 (IRE1), PERK, and activating transcription factor 6 (ATF6) (Gregor and Hotamisligil, 2007; Walter and Ron, 2011).

In our study, HFD-fed rats exhibited significantly increased expression of PERK and its downstream target eIF2α, alongside elevated phosphorylation of both proteins, indicative of UPR activation. This finding aligns with previous studies that link ER stress with hepatic steatosis and insulin resistance, hallmarks of MASLD (Jo et al., 2013; Venkatesan et al., 2023; Zhao et al., 2013).

Notably, SREBP1c activation under ER stress can occur independently of insulin signaling, suggesting that stress responses may drive lipogenesis even in insulin-resistant states (Mak et al., 2023; Su et al., 2020). Moreover, SREBP1c overexpression has been shown to exacerbates ER stress, creating establishing a feedback loop that further amplifies lipid accumulation and hepatocellular injury (Hu et al., 2020).

Under conditions of acute stress, the UPR supports autophagy to degrade misfolded proteins and lipid droplets. However, during prolonged or chronic ER stress, as induced by sustained HFD intake, autophagy is suppressed, and apoptosis is triggered (Guo and Li, 2014). In our HFD-fed rats, key autophagy markers, including LC3B, Beclin-1, ATG16L1, and ATG9A, were significantly downregulated, while p62, a marker of impaired autophagy, was upregulated. Concurrently, apoptosis markers, such as Caspase 3, were elevated, while anti-apoptotic BCL-2 was downregulated. These findings indicate a pathological shift from protective autophagy to programmed cell death during chronic lipid overload.

*In vitro* experiments using HepG2 cells treated with fatty acids showed similar downregulation of miR-18a-5p and upregulation of SREBP1c, reinforcing the animal model findings. The observation that FAs treatment did not affect cell viability but promoted lipid accumulation supports the utility of this model for mechanistic studies on MASLD (García-Ruiz et al., 2015; Gómez-Lechón et al., 2007). Moreover, the observed impairment in autophagic flux and increased apoptosis in FA-treated HepG2 cells further illustrate the detrimental effects of lipid overload on hepatocyte function (Lee et al., 2018). Crucially, overexpression of miR-18a-5p in HepG2 cells reduced lipid accumulation, mitigates ER stress, attenuated apoptotic activity, and improved the autophagic flux. These results highlight the protective role of miR-18a-5p against the cellular effect of lipotoxicity and suggest that restoring its levels could be a viable strategy to counteract MASLD. While the combined *in vivo*/*in vitro* approach represents a strength of the manuscript, its generalizability may be limited. Further research will focus on a more in-depth investigation of miRNA-target interactions and pathway cross-talk. Moreover, *in vivo* miRNA overexpression is still lacking. In addition, given the complexity of liver physiology, exploring the crosstalk between miR18a-5p signaling and other relevant pathways would be worthwhile. In conclusion this study identifies miR-18a-5p as a key modulator of hepatic lipid metabolism, ER stress, autophagy, and apoptosis in the setting of HFD-induced MASLD. Restoring miR-18a-5p levels may represent a promising therapeutic approach to reduce fat accumulation, alleviate ER stress, and protect against hepatocyte damage in MASLD.

## Data Availability

The raw data supporting the conclusions of this article will be made available by the authors, without undue reservation.
