# Correction: MicroRNA-18a-5p regulates hepatic lipid accumulation in response to high-fat diet

**DOI:** 10.3389/fphys.2025.1706519

**Published:** 2025-09-24

**Authors:** Giuseppe Petito, Nunzia Magnacca, Arianna Cuomo, Maria Ventriglia, Angelo Fusco, Massimo Venditti, Sara Falvo, Nicoletta Potenza, Antonia Lanni, Federica Cioffi, Rosalba Senese

**Affiliations:** ^1^ Department of Environmental, Biological and Pharmaceutical Sciences and Technologies, University of Campania “Luigi Vanvitelli”, Caserta, Italy; ^2^ Department of Experimental Medicine, University of Campania “Luigi Vanvitelli”, Naples, Italy; ^3^ Department of Science and Technologies, University of Sannio, Benevento, Italy

**Keywords:** miRNA, ER stress, autophagy, apoptosis, fats, liver, triglyceride

The published article, the funder [Italian Ministry of University and Research (PRIN2022)], [CUP: B53D23011470006, Project number: 2022XHXE4E] was erroneously omitted. The corrected **Funding** statement appears below.

