# Supplementary material for: MicroRNA-18a-5p regulates hepatic lipid accumulation in response to high-fat diet
Source: Front Physiol. 2025 Sep 4;16:1661428. doi: 10.3389/fphys.2025.1661428 (PMC12443723; doi:10.3389/fphys.2025.1661428)
Supplement: Supplementary file 1 [file Supplementaryfile1.pdf]

Supplementary Materials

1 Supplementary Figures and Tables

1.1 Supplementary Figure

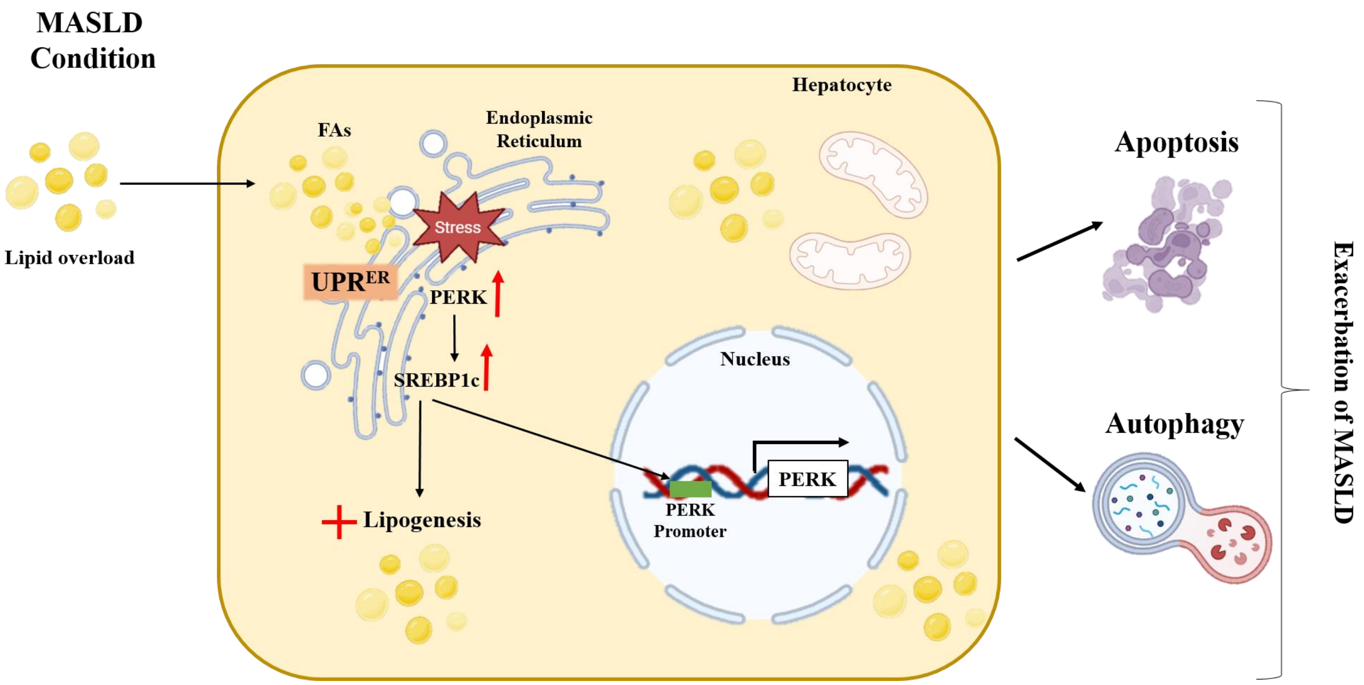

Supplementary Figure 1. Schematic representation of the studied pathway involving SREBP1, ER, and UPR

1.2 Supplementary Tables

Supplementary Table 1. Antibodies

**Antibody**	Reference number
SREBP1c	Santa Cruz Biotechnology -cod.sc-366
p-PERK(Thr980)	Cell Signaling -cod.#3179
PERK	Cell Signaling -cod.#3192
p-eIF2α(Ser51)	Cell Signaling -cod.#3398
eiF2α	Cell Signaling -cod.# 2103
ATF4	Abclonal -cod.#A18687
LC3B	Santa Cruz Biotechnology -cod.sc-376404
ATG5	Novus Biologicals -cod. NB110-53818
ATG16L1	Novus Biologicals -cod. NB110-82384
ATG9A	Novus Biologicals -cod. NB110-56893
Beclin-1	Cell Signaling -cod.# 3738
p62	Cell Signaling -cod.#5114
Caspase-3	Cell Signaling -cod.#9662
Bcl-2	Santa Cruz Biotechnology -cod.sc-7382
B-ACTIN	Bioss Antibodies -cod.bs-0061R
